# Establishing a hospital transfusion management system promotes appropriate clinical use of human albumin in Japan: a nationwide retrospective study

**DOI:** 10.1186/s12913-019-4836-0

**Published:** 2019-12-26

**Authors:** Yoshiteru Yano, Nobuo Sakata, Kiyohide Fushimi

**Affiliations:** 1 Department of Health Policy and Informatics, Tokyo Medical and Dental University Graduate School, 1-5-45 Yushima, Bunkyo-ku, Tokyo, 113-8510 Japan; 2 Department of Health Services Research, University of Tsukuba, 1-1-1 Tennodai, Tsukuba, Ibaraki, 305-8575 Japan

**Keywords:** Transfusion medicine, Serum albumin, Practice management, Hospital records, Big data

## Abstract

**Background:**

Despite international recommendations to establish hospital transfusion management systems to promote appropriate use of blood products, the general efficacy of establishing such systems has not been proven. This study aimed to validate the effect of establishing such systems for promoting the appropriate use of human albumin.

**Methods:**

In this retrospective observational study, we used a Japanese Diagnosis Procedure Combination (DPC) database from fiscal year 2012 to 2016, which included inpatient records from approximately 1200 hospitals for payment processes in the national medical insurance system. From this existing database, containing approximately 8 million inpatient records per year, we selected patients with emergency due to “bleeding,” “sepsis,” and “burn injury,” by using the International Classification of Diseases and Injuries 10th revision (ICD-10) codes, and hospitals that had one or more patients for each disease group in each fiscal year. We conducted multivariable logistic regression analysis to estimate the relationship between human albumin administration and the state of the hospital transfusion management system. We evaluated temporal trends of mortality rate and length of stay as an indicator of care quality.

**Results:**

Overall, 139,853 eligible patients admitted to 682 hospitals were selected. The results of the multivariable logistic regression analysis show that patients who were admitted to hospitals with an established hospital transfusion department introducing good practice criteria of blood products were less likely to be administered human albumin compared with those who were admitted to hospitals not introducing it, by approximately 30% for each of the three disease groups; adjusted odds ratios (95% confidential intervals) were 0.70 (0.59–0.83), 0.75 (0.69–0.81), and 0.71 (0.58–0.87) in the “bleeding,” “sepsis,” and “burn injury” groups, respectively. The temporal trends evaluation shows that there were no increasing trends of mortality rate and average length of stay against decreasing trends of human albumin administration in any disease groups.

**Conclusions:**

Establishing a hospital transfusion department responsible for promoting appropriate clinical use of blood products could reduce human albumin administration for critically ill patients without loss of care quality. These findings provide support for policy makers and hospital managers to consider establishing such systems.

## Background

Blood products have high efficacy for specific patients when used correctly [1]. However, as blood products are a scarce and expensive human resource, they should only be administered to treat conditions associated with significant morbidity or mortality that cannot be prevented or managed effectively by other means [1]. Appropriate clinical use of blood products has been recognized as an important issue internationally, including timely availability for all patients when required, ensuring global self-sufficiency and eliminating involuntary blood donations [2, 3].

Human albumin (HA) is a blood product made of plasma proteins that has varying indications, including for conditions such as liver cirrhosis or for therapeutic plasma exchange. The promotion of the appropriate clinical use of HA is important, because inappropriate clinical use of HA, such as for hypoalbuminemia and nutrition supply, has been reported in many countries [4, 5]. According to previous studies, 40 to 90% of HA administration was not supported by clinical guidelines at the time [4, 6, 7]. Furthermore, HA consumption per 1 million population has increased in several developed countries, such as the United States, Italy, and France [5, 8]. In Japan, HA consumption per 1 million population has decreased, but the domestic self-sufficiency rate of HA remains less than 60% and has not improved [8]. As the worldwide demand for HA is expected to increase in the future [9], it is apparent that implementing clinical guidelines for appropriate use of HA in each hospital has become more important.

Clinical studies evaluating HA efficacy report no clear benefit of HA compared to alternatives, such as crystalloids or non-protein colloids, for initial resuscitation in critically ill patients, such as patients with “bleeding,” “sepsis,” and “burn injury” [4, 10–12]. As such, worldwide clinical guidelines for appropriate use of HA now specify that HA administration is not recommended for initial resuscitation in these critically ill patients, except in special circumstances [13–16]. In Japan, clinical guidelines with similar recommendations were released in June 2015 by the Japan Transfusion and Cell Therapy Association [17].

In 2003, the World Health Organization recommended that national and hospital authorities allocate sufficient resources to implement clinical guidelines on the appropriate use of blood products, such as a hospital transfusion management system responsible for implementation and monitoring practices [1, 18, 19]. According to these international recommendations, the Japanese government of Ministry of Health, Labour and Welfare (MHLW) issued hospital guidelines for implementing transfusion medicine and set financial incentives on the national medical insurance system for all hospitals conducting blood transfusion to comply with the hospital guidelines [20].

However, to the best of our knowledge, previous studies evaluating the effect of establishing a hospital transfusion management system on promoting the appropriate clinical use of blood products were limited to case reports conducted in only 1 or few hospitals [21–25]. Therefore, the general efficacy of establishing such system has not been proven. The purpose of this study was to conduct a quantitative nationwide retrospective evaluation to validate whether establishing a hospital transfusion management system is generally effective for promoting the appropriate use of HA.

## Methods

### Study design and setting

Almost all medical services provided in Japanese hospitals are reimbursed to hospitals based on the medical fee schedule table from the national medical insurance system covering all citizens in Japan. As the medical fee schedule table is uniform across the country and hospitals are prohibited in principle to claim an additional fee to patients and insurers that is not on the medical fee schedule table on their own, the prices of the medical fees paid from the insurer to hospitals are the same across all Japanese hospitals, with some exceptions. The medical schedule table is defined by the MHLW and revised every 2 years in line with the latest progress in medical technology. As the MHLW has set an additional fee in the medical fee schedule table as an incentive to comply with various guidelines, a compliant hospital can gain an additional fee from the national medical insurer. The hospital guidelines on implementing transfusion medicine issued by the MHLW recommend that all hospitals conducting blood transfusion establish a hospital transfusion management system; therefore, an additional fee to comply with these guidelines was set in the medical fee schedule.

According to the survey conducted in fiscal year (FY) 2016 of the hospitals that had performed blood transfusions in Japan, among 3681 hospitals replying, 1729 (47%) had established a hospital transfusion management system to receive the additional fee, and more than 90% of human albumin products were consumed in hospitals that had established a hospital transfusion management system [26].

### Data source

This retrospective observational study used data obtained from the Japanese Diagnosis Procedure Combination (DPC) database from fiscal year (FY) 2012 to 2016 (i.e., from April 2012 to March 2017), which was created for the purpose of evaluating functions and roles of hospitals. DPC data included nationwide inpatient records created by acute-care hospitals for claim and payment processes in the national medical insurance system mentioned above. This existing database contains approximately 8 million DPC data per year submitted from approximately 1200 voluntary participating hospitals, covering approximately 50% of all admissions to acute-care hospitals in Japan. Details of the DPC data have been mentioned elsewhere [27, 28]. We obtained baseline patient information, such as age, sex, diagnosis coded by the International Classification of Diseases and Injuries 10th revision (ICD-10), and consciousness level at admission from the DPC database. We also obtained the medical procedural information from each patient during the entire hospitalization, such as mechanical ventilation and HA administration.

### Data selection procedures

From the DPC database, we selected patients aged 15 years or older who were admitted via emergency to acute-care wards because of “bleeding,” “sepsis,” or “burn injury.” Patients who were admitted via emergency were identified by baseline patient information as follows: admission type was not scheduled or admission via ambulance service. The “bleeding” group included patients whose diagnosis causing hospitalization was traumatic or obstetric bleeding, identified by the following ICD-10 codes: J942; S15; S25; S26; S27–29; S35–39; S45; S48; S55; S58; S65; S68; S75; S78; S85; S88; S95; S98; T05; T063; T065; T096; T114; T116; T134; T136; T145; T147; T792; T794; O031; O036; O041; O046; O051;O056; O061; O066; O071; O076; O081; O201; O208; O209; O441; O45; O46; O67; O71; O720; O721; O722; O902. The “sepsis” group included patients whose diagnosis that caused hospitalization or co-morbidity at admission was sepsis, identified by the following ICD-10 codes: A021; A327; A39; A40; A41; B007; B250; B252; B376; B377; B387; B393; B407; B417; B427; B447; B464. The “burn injury” group included patients whose diagnosis that caused hospitalization was burn injury, identified by the following ICD-10 codes: T200–203; T210–213; T220–223; T230–233; T240–243; T250–253; T260–264; T270–273; T280–284; T290–293; T300–303; T31. We selected hospitals that had one or more patients for each condition in each fiscal year. Therefore, the observed hospitals were fixed during all observed fiscal years in this study.

To exclude patients for whom HA administration may have been appropriate, we excluded patients with comorbidities for which HA administration was recommended by the guidelines [17] as follows: (1) liver cirrhosis (K702–704; K717; K720; K721; K740–746; K766; K767; R18), congestive heart failure (I110; I130; I50), renal failure (E102; E112; E122; E132; E142; I120; N17; N18; N19; N990; O904), and neuromuscular diseases (G61; G70). We excluded patients under exceptional conditions as follows: (2) underwent transplantation surgery; (3) involved in clinical research; and (4) death within 24 h from admission.

### Data measurement

We obtained information about the state of the hospital transfusion management system of each hospital by using medical procedural codes associated with the medical fees paid for complying with the hospital guidelines on implementing transfusion medicine, as follows.

“Hospital transfusion department (HTD)”: A medical fee was paid for hospitals with an HTD meeting the conditions recommended in the hospital guidelines, as an incentive to promote safe and appropriate transfusion. In brief, the conditions were as follows: First, the HTD must consist of 1 or more full-time medical doctors responsible for all transfusion procedures in the hospital and 1 or more full-time clinical laboratory technicians, with all tasks associated with blood transfusion carried out centrally, such as blood transfusion-related examination, billing, storage, and dispensing of blood products. Second, the hospital transfusion committee (HTC), consisting of a hospital administrator and other medical staff associated with transfusion therapy to monitor the blood products practices in the hospital, must meet periodically, at least 6 times per year [20, 29].

“Good practice criteria (GPC) of blood products”: An additional fee was paid for hospitals with an HTD mentioned above introducing good practice criteria of blood products as an incentive to promote implementation of clinical guidelines of blood products. In brief, the criteria was as follows: the units of HA used throughout the hospital excluding plasma exchange therapy, which is estimated by dividing the amount of used HA (g) by 3, was less than 2 times the units of used red blood cell concentrates [29].

The previous survey in Japan reported that within relatively large hospitals with 300 or more beds, which consumed approximately 83% of all blood products in Japan, as much as 91% hospitals had an HTD, with only 64% of all hospitals introducing GPC of blood products to an HTD [26]. In this study, in order to assess the effect of introduction of GPC to an HTD, we categorized the state of the hospital transfusion management system into 3 hospital groups, as follows: “HTD introducing GPC” where hospitals had an HTD and introduced GPC of blood products, “HTD not introducing GPC” where hospitals had an HTD but did not introduce GPC of blood products, and “non-HTD” where hospitals did not have an HTD.

The primary outcome was HA administration as a binary variable. If a patient was administered 1 or more units of HA during hospitalization, we regarded the patient as administered HA. This was identified using medical procedural codes. The secondary outcomes were in-hospital mortality rate and average hospital length of stay (LOS) for each fiscal year, which could be identified from baseline patient information. These outcomes were estimated as an indicator to evaluate care quality as was done in previous studies [10–12].

Potential confounding factors were identified, such as age, level of consciousness at admission, Charlson Comorbidity Index (CCI), and mechanical ventilation. These covariates were selected based on a previous study [30]. Patient age was categorized as 15–39, 40–69, and ≥ 70 years. Level of consciousness at admission was assessed using the Japan Coma Scale (JCS) score as follows: 0 (alert), 1–3 (delirious), 10–30 (somnolent), and 100–300 (comatose) [31]. The Sundararajan version of the CCI at admission was calculated based on ICD-10 diagnosis for comorbidities, and categorized as 0, 1, 2, and ≥ 3 [32]. We identified the Burn Index in the “burn injury” group from baseline patient information. We identified potential confounding hospital characteristics, including academic status (academic or non-academic) and the number of beds. These covariates were selected based on a previous study evaluating the care quality of multiple hospitals [27, 33]. Number of beds was categorized as “less than 200 (small size),” “greater than or equal to 200 and less than 500 (middle size),” and “more than or equal to 500 (large size).”

### Data analysis

We evaluated temporal trends of each of the 3 outcomes mentioned above (i.e. HA administration, mortality rate, and LOS) for the 3 disease groups, as follows: the Cochran-Armitage test was used to evaluate the proportion of HA administration and in-hospital mortality rate for each fiscal year as categorical variables, and Spearman’s rank correlation was used to evaluate the average hospital LOS for each fiscal year as continuous variables. Furthermore, we evaluated temporal trends of in-hospital mortality rate and average hospital LOS for each fiscal year using the same statistical analysis for each of the 6 subgroups, defined by each of the 3 disease groups (bleeding, sepsis and burn injury) in the 2 hospital groups (i.e. HTD introducing GPC, HTD not introducing GPC) to validate whether the introduction of GPC of blood products to an HTD had had a positive or negative influence on care quality.

We conducted multivariable logistic regression analysis for each of the 3 disease groups independently to evaluate relationships between HA administration and the state of the hospital transfusion management system. We used the multivariable logistic regression model with generalized estimating equations to consider clustering of patients within hospitals because the data were collected from multiple hospitals [34]. We estimated the adjusted odds ratio of HA administration of the “HTD introducing GPC” group with reference to the “HTD not introducing GPC” group to validate quantitatively the effect of the introduction of GPC of blood products to an HTD and the “non-HTD” group with reference to the “HTD not introducing GPC” group to validate the effect of establishing an HTD itself. Multicollinearity between independent variables was assessed by estimating variance inflation factors (reference value of 10) before estimating the final output.

Patients with missing values were excluded from the analysis. The threshold for significance was *P* < 0.05. All statistical analyses were performed in STATA version 14.0 (Stata Corp, College Station, TX, USA).

## Results

### Patient selection and characteristics

We identified 139,853 eligible patients admitted to 682 eligible hospitals. Baseline patient characteristics for each of the 3 hospital groups of the state of hospital transfusion management system are presented in Table 1. The total numbers of patients in each disease group were as follows: 21,577 in the “bleeding” group, 110,462 in the “sepsis” group, and 7814 in the “burn injury” group. The group “HTD introducing GPC” contained 97,956 patients, of whom 17,117 (17.5%) were administered HA. The group “HTD not introducing GPC” contained 36,484 patients, of whom 9320 (25.5%) were administered HA. The group “non-HTD” contained 5413 patients, of whom 1036 (19.1%) were administered HA.

**Table 1:** Baseline patient characteristics for each of the 3 hospital groups

	HTD introducing GPC (*N* = 97,956)		HTD not introducing GPC (*N* = 36,484)		Non-HTD (*N* = 5413)		Total (*N* = 139,853)	
	N	%	N	%	N	%	N	%
Age, years								
15–39	10,380	10.6	5521	15.1	450	8.3	16,351	11.7
40–69	23,781	24.3	10,069	27.6	1306	24.1	35,156	25.1
≥ 70	63,795	65.1	20,894	57.3	3657	67.6	88,346	63.2
Sex								
Male	51,128	52.2	18,796	51.5	2852	52.7	72,776	52.0
Diagnosis								
Bleeding	13,600	13.9	7216	19.8	761	10.4	21,577	15.4
Sepsis	79,500	81.2	26,638	73.0	4324	79.9	110,462	79.0
Burn injury	4856	5.0	2630	7.2	328	6.1	7814	5.6
Conscious level at admission (JCS)								
0 (alert)	56,672	59.2	21,641	60.7	2607	50.5	80,920	57.9
1–3 (delirious)	22,632	23.6	7955	22.3	1453	28.2	32,040	22.9
10–30 (somnolent)	8800	9.2	3154	8.8	597	11.6	12,551	9.0
100–300 (comatose)	7686	8.0	2924	8.2	501	9.7	11,111	7.9
Charlson comorbidity index								
0	51,397	52.5	20,661	56.6	2870	53.0	74,928	53.6
1	23,519	24.0	8080	22.1	1371	25.3	32,970	23.6
2	13,712	14.0	4563	12.5	709	13.1	18,984	13.6
≥ 3	9328	9.5	3180	8.7	463	8.6	12,971	9.3
HA administration								
Administered	17,117	17.5	9320	25.5	1036	19.1	27,473	19.6
Mechanical ventilation								
Conducted	7695	7.9	3683	10.1	376	6.9	11,754	8.4
In-hospital death								
Death	16,029	16.4	5435	14.9	966	17.8	22,430	16.0
Hospital LOS (mean, SD)	(26.9, 30.7)		(27.7, 32.7)		(32.3, 40.1)			
Admitted hospital type								
Academic	12,424	12.7	12,113	33.2	38	0.7	24,575	17.6
Admitted hospital bed size								
< 200	3046	3.1	1445	4.0	948	17.5	5439	3.9
200–499	34,304	35.0	16,055	44.0	3294	60.9	53,653	38.4
≥ 500	60,606	61.9	18,984	52.0	1171	21.6	80,761	57.7

*GPC* good practice criteria of blood products; *HA* human albumin; *HTD* hospital transfusion department; *JCS* Japan Coma Scale; *LOS* length of stay; *SD* standard deviation

### Temporal trend of hospital and patient characteristics

The temporal trend of the number of hospitals in each of the 3 hospital groups is presented in Table 2. The decreasing rate of the proportion of HA administration to total patients (%) from FY2012 to FY2016 (estimated by dividing the difference between the value of FY2012 and FY2016 by the value of FY2012) and the *P* value of the Cochran-Armitage test of it for each of the 3 disease groups across all hospitals were as follows: 18.7% (from 10.7 to 8.7; *P* = 0.002) in the “bleeding” group, 9.6% (from 22.9 to 20.7; *P* < 0.001) in the “sepsis” group, and 10.5% (from 25.7 to 23.0; *P* = 0.089) in the “burn injury” group. The decreasing rate of the in-hospital mortality rate and the *P* value of the Cochran-Armitage test of it were as follows: 0.0% (from 1.4 to 1.4; *P* = 0.60) in the “bleeding” group, 15.0% (from 22.0 to 18.7; *P* < 0.001) in the “sepsis” group, and 15.8% (from 7.6 to 6.4; *P* = 0.28) in the “burn injury” group. The decreasing rate of the average hospital LOS and the *P* value of the Spearman’s rank correlation of it were as follows: 14.5% (from 19.3 to 16.5; *P* = 0.037) in the “bleeding” group, 10.1% (from 30.6 to 27.5; *P* < 0.001) in the “sepsis” group, and 8.3% (from 36.2 to 33.2; *P* = 0.037) in the “burn injury” group. In summary, declining trends or flat trends of absolute values were observed in the proportion of HA administration, in-hospital mortality rate, and average hospital LOS in each of the 3 disease groups; no significant increasing trend was observed.

**Table 2:** Temporal trends in the number of hospitals and patient outcomes

	FY 2012	FY 2013	FY 2014	FY 2015	FY 2016	*P* value
*Hospital characteristics (Total No.: 682)*						
HTD introducing GPC	418	418	454	454	451	
HTD not introducing GPC	187	187	172	172	177	
Non-HTD	77	77	56	56	54	
*Patient characteristics*						
“Bleeding” total patients No.	3928	4331	4487	4357	4474	
HA administration No.	419	432	414	367	390	
Prop. (%)	10.7	10.0	9.2	8.4	8.7	0.002^a^
In-hospital death No.	54	57	48	62	62	
Mortality rate (%)	1.4	1.3	1.1	1.4	1.4	0.60^a^
Average hospital LOS (day)	19.3	19.1	17.7	16.3	16.5	0.037†
“Sepsis” total patients No.	18,406	19,973	23,301	24,452	24,330	
HA administration No.	4209	4413	4933	4937	5028	
Prop. (%)	22.9	22.1	21.2	20.2	20.7	< 0.001^a^
In-hospital death No.	4052	4008	4515	4425	4543	
Mortality rate (%)	22.0	20.1	19.4	18.1	18.7	< 0.001^a^
Average hospital LOS (day)	30.6	30.3	28.1	27.6	27.5	< 0.001†
“Burn injury” total patients No.	1598	1675	1647	1531	1363	
HA administration No.	410	446	406	356	313	
Prop. (%)	25.7	26.6	24.7	23.3	23.0	0.089^a^
In-hospital death No.	122	142	132	121	87	
Mortality rate (%)	7.6	8.5	8.0	7.9	6.4	0.28^a^
Average hospital LOS (day)	36.2	35.4	33.6	33.7	33.2	0.037†

Note: Mortality rate was estimated by dividing “in-hospital death No.” by “Total patient No.”. The results of the statistical analysis of patient outcomes were estimated for each of the 3 patient groups across all hospitals. ^a^Obtained by the Cochran-Armitage test; †Obtained by Spearman’s rank correlation. *HA* human albumin; *FY* fiscal year (begin on 1 April of the year, and end on 31 March of the next year); *HTD* hospital transfusion department; *GPC* good practice criteria of blood products; *Prop* proportion

### Care quality

**Fig. 1:**
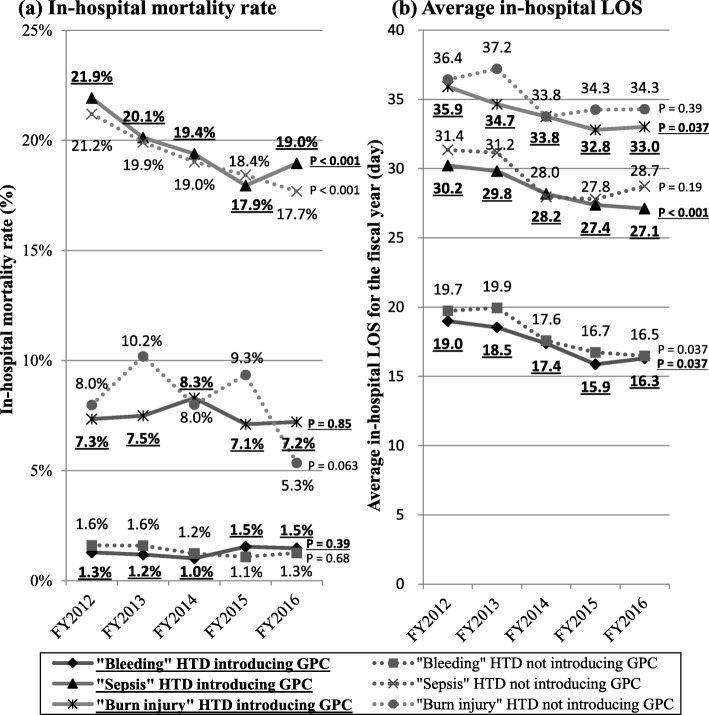
**a-b** Temporal trend of: **a** Mortality rate, and **b** Average length of stay (LOS) in the 6 subgroups. The subgroups were defined as each of the 3 disease groups of “bleeding,” “sepsis,” and “burn injury” in the 2 hospital groups of “HTD introducing GPC” and “HTD not introducing GPC.” *P* value was obtained by the Cochran-Armitage test in Fig. 1a and by Spearman’s rank correlation in Fig. 1b. FY, fiscal year (beginning on the April 1 and ending on March 31 of the next year); HTD, hospital transfusion department; GPC, good practice criteria of blood products; LOS, length of stay

The results of the statistical analysis for the temporal trend of in-hospital mortality rate and average hospital LOS for each fiscal year in the 6 subgroups, defined as each of the 3 disease groups in the 2 hospital groups of “HTD introducing GPC” and “HTD not introducing GPC,” are presented in Fig. 1. As shown in Fig. 1-a, the change in the rate of in-hospital mortality rate of each of the 3 disease groups from FY2012 to FY2016 (estimated by dividing the difference between the value of FY2012 and FY2016 by the value of FY2012) and the *P* value of the Cochran-Armitage test of it were as follows: “bleeding” group: 15.3% increase (from 1.3 to 1.5, *P* = 0.39) in “HTD introducing GPC” group, 18.8% decline (from 1.6 to 1.3; *P* = 0.68) in “HTD not introducing GPC” group; “sepsis” group: 13.2% decline (from 21.9 to 19.0; *P* < 0.001) in “HTD introducing GPC” group, 16.5% decline (from 21.2 to 17.7; P < 0.001) in “HTD not introducing GPC” group; “burn injury” group: 1.4% decline (from 7.3 to 7.2; *P* = 0.85) in “HTD introducing GPC” group, 33.8% decline (from 8.0 to 5.3; *P* = 0.063) in “HTD not introducing GPC” group. As shown in Fig. 1-b, the change in the rate of average hospital LOS and the *P* value of the Spearman’s rank correlation of it were as follows: “bleeding” group: 14.2% decline (from 19.0 to 16.3; *P* = 0.037) in “HTD introducing GPC” group, 16.2% decline (from 19.7 to 16.5; *P* = 0.037) in “HTD not introducing GPC” group; “sepsis” group: 10.3% decline (from 30.2 to 27.1; *P* < 0.001) in “HTD introducing GPC” group, 8.6% decline (from 31.4 to 28.7; *P* = 0.19) in “HTD not introducing GPC” group; “burn injury” group: 8.1% decline (from 35.9 to 33.0; *P* = 0.037) in “HTD introducing GPC” group, 5.8% decline (from 36.4 to 34.3; *P* = 0.39) in “HTD not introducing GPC” group. In summary, the presence or absence of a statistically significant difference between the 2 hospital groups are either the same, or present only in “HTD introducing GPC” in each of the 3 disease groups.

### Effect of hospital transfusion management system on clinical use of human albumin

As shown in Table 3, the results of the study indicated that HA administration for each of the 3 disease groups in patients in the “HTD introducing GPC” group, with reference to patients in “HTD not introducing GPC” group, was significantly less likely. The statistically significant difference was 30, 25, and 29%, in the “bleeding” group, the “sepsis” group, and the “burn injury” group, respectively. Adjusted odds ratios (95% confidential intervals) were 0.70 (0.59–0.83) in the “bleeding” group, 0.75 (0.69–0.81) in the “sepsis” group, and 0.71 (0.58–0.87) in the “burn injury” group (Table 3). On the other hand, HA administration in patients in the “non-HTD” group, with reference to patients in the “HTD not introducing GPC” group, was not significantly different in the 3 disease groups; adjusted odds ratios were 0.81 (0.52–1.24) in the “bleeding” group, 1.12 (0.95–1.32) in the “sepsis” group, and 0.65 (0.38–1.12) in the “burn injury” group.

**Table 3:** Multivariable logistic regression analysis of HA administration for each of the 3 disease groups

	Bleeding	Sepsis	Burn injury
	OR (95% CI)	OR (95% CI)	OR (95% CI)
Age			
15–39	Ref.	Ref.	Ref.
40–69	0.78 (0.68–0.88)	1.85 (1.66–2.06)	2.31 (1.82–2.95)
≥ 70	1.22 (1.05–1.42)	1.45 (1.30–1.61)	5.98 (4.68–7.63)
JCS			
0 (alert)	Ref.	Ref.	Ref.
1–3 (delirious)	3.96 (3.41–4.59)	1.13 (1.08–1.17)	1.97 (1.65–2.35)
10–30 (somnolent)	6.41 (5.22–7.86)	1.36 (1.29–1.43)	2.90 (2.08–4.05)
100–300 (comatose)	10.52 (8.66–12.79)	1.78 (1.69–1.88)	4.23 (3.16–5.66)
CCI			
0	Ref.	Ref.	Ref.
1	1.12 (0.92–1.36)	0.92 (0.88–0.95)	1.51 (1.27–1.81)
2	1.13 (0.77–1.64)	0.93 (0.89–0.97)	1.44 (1.08–1.92)
≥ 3	1.50 (0.88–2.54)	0.85 (0.81–0.90)	1.32 (0.85–2.05)
Burn index			
< 10			Ref.
≥ 10			12.25 (10.59–14.17)
Mechanical ventilation			
Not performed	Ref.	Ref.	Ref.
Performed	12.97 (6.77–24.85)	5.50 (5.25–5.76)	10.00 (6.78–14.75)
Hospital type			
Non-academic	Ref.	Ref.	Ref.
Academic	1.54 (1.21–1.94)	2.04 (1.69–2.46)	1.84 (1.41–2.41)
Hospital bed size			
< 200	Ref.	Ref.	Ref.
200–499	1.64 (0.85–3.16)	1.50 (1.25–1.81)	2.98 (1.31–6.75)
≥ 500	2.22 (1.15–4.27)	1.89 (1.54–2.32)	5.01 (2.21–11.31)
Hospital transfusion management system			
HTD not introducing GPC	Ref.	Ref.	Ref.
HTD introducing GPC	0.70 (0.59–0.83)	0.75 (0.69–0.81)	0.71 (0.58–0.87)
Non-HTD	0.81 (0.52–1.24)	1.12 (0.95–1.32)	0.65 (0.38–1.12)

*CCI* Charlson comorbidity index; *CI* confidence intervals; *GPC* good practice criteria of blood products; *HA* human albumin; *HTD* hospital transfusion department; *OR* odds ratio; *Ref* reference

## Discussion

In this study we used nationwide inpatient record data to evaluate the effect of the state of hospital transfusion management system on both HA administration and care quality. Our results showed that establishing an HTD introducing GPC of blood products could reduce HA administration for critically ill patients without loss of care quality.

Similar to the purpose of our study, several previous studies have evaluated the effectiveness of various interventions conducted by hospital transfusion management systems to promote appropriate use of blood products. Studies conducted in 1 hospital in Iran [22], 1 hospital in Italy [23], and 2 hospitals in Italy [24] reported that the implementation of hospital guidelines on the clinical use of HA performed by some kinds of hospital transfusion management system could reduce HA consumption by 36%, 15–20%, and 7.6–77.4%, respectively. Other studies conducted in 1 hospital in India [21] and 1 hospital in Portugal [25] reported that an educational intervention conducted by the hospital transfusion management system may be an effective measure to promote appropriate clinical use of blood products. These previous studies revealed the effectiveness of hospital central management of transfusion in promoting appropriate use of blood products, so they were consistent with our study, and also with international recommendations to establish hospital transfusion management systems responsible for promoting appropriate use of blood products.

Our study has greater strength compared to these previous studies, because we used big data from 682 acute-care hospitals rather than only 1 or a few hospitals. This makes it possible to assess the general efficacy of establishing an HTD. A further strength of our study was that we used patient-record data rather than whole hospital performance data, such as total number of patients a year, total usage of HA a year, etc. Using patient-record data makes it possible to assess the effect of an HTD precisely by adjusting for patient characteristics that were different in each hospital. For example, a survey conducted by the MHLW on the implementation status of blood transfusion targeted more hospitals than our study; however, the survey only included a questionnaire about whole hospital performance and did not use a patient-record data [26]. Contrary to the survey, our study could adjust for patient characteristics such as sex, age, and disease severity, by conducting multivariable logistic regression analysis using patient-record data. Therefore, we could evaluate the efficacy of establishing an HTD quantitatively and with greater accuracy by each disease.

As shown in Table 2, the proportion of HA administration to total patients tended to decrease in each disease group from FY2012 to FY2016. According to the survey conducted by the MHLW in Japan, the total HA consumption, in terms of the raw material, has decreased by approximately 23% from 150.4 kL in 2010 to 114.9 kL in 2015 [8]; therefore, our results were roughly consistent with their results from the view of a decreasing trend in HA consumption. We also examined the in-hospital mortality rate and average hospital LOS for each fiscal year as an indicator of care quality. As shown in Table 2, there was no clear evidence that the care quality declined against the decrease in HA administration. These results suggest that the decreasing trend in HA administration is related to an advancement in more appropriate clinical use of HA, not a decline in required medical service. To the best of our knowledge, the results are unprecedented, because ours is the first nationwide evaluation conducted in each critically ill patient group to validate the temporal trends of both the proportion of HA administration and care quality.

Results of multivariable logistic regression analysis showed that HA administration in patients in the “HTD introducing GPC” group with reference to patients in the “HTD not introducing GPC” group was fewer by approximately 30% for each of the 3 disease groups. Furthermore, as shown in Fig. 1, there was no clear evidence that the introduction of GPC of blood products to an HTD caused a decline in care quality. These results suggest that the introduction of GPC of blood products in hospitals with an HTD could reduce HA administration without loss of care quality in all critically ill patients. On the contrary, this result also suggests many HTDs were not sufficient to address the appropriate clinical use of blood products. Several surveys have reported that some parts of established hospital transfusion management systems do not make enough effort to improve clinical use of blood products; a previous survey conducted in 121 hospitals worldwide reported a large variation in the structure and activity of HTCs, including the consisting members and the frequency of meetings, and many HTCs did not report important quality variables associated with transfusion management [35]. Furthermore, the survey conducted in Japan reported that more than 45% of hospitals answered that promotion of appropriate clinical use of blood products was left to the efforts of individual clinical doctors, which means that no activity to improve clinical use of blood products was conducted by an HTD [26]. Our results are consistent with these previous surveys; in our study, 177 hospitals with an HTD not introducing GPC of blood products in FY 2016, which account for 28% of all 628 hospitals with an HTD, seem not have made enough effort to promote appropriate use of HA compared with hospitals with an HTD introducing GPC. Considering the fact that the number of hospitals with an HTD introducing GPC of blood products has remained at only approximately 30% of all hospitals conducting blood transfusions in Japan at the time of FY2016 [26], it appears that there is room for increased promotion of the appropriate use of HA by additional efforts.

Our study has several limitations. First, we lacked data regarding vital signs from each patient, such as serum albumin, blood pressure, urine volume, and disease severity, such as the amount of blood loss in the “bleeding” group and the sequential organ failure assessment score in “sepsis” patients. Therefore, we could not assess the appropriateness of HA administration based on the precise clinical status of each patient. Instead, we assessed whether the decreasing trend of HA administration means an advancement in the appropriate clinical use or there is simply a decline in required medical services, by using in-hospital mortality and LOS. Second, we lacked precise information regarding the activities conducted by the HTD for promoting appropriate clinical use of HA; therefore, we could not assess the precise reason why HTDs introducing GPC of blood products achieved lower administration of HA. Further study is needed on what types of whole-hospital interventions, such as education or an audit, should be conducted by the HTD to implement clinical guidelines. Third, there may be unknown hospital factors confounded with establishing an HTD. Therefore, in our study, we could not conclude that the lower proportion of HA administration was caused by establishing an HTD. Finally, this may not be representative of all patients, as not all acute-care hospitals in Japan submitted records to the DPC database.

## Conclusions

Our study suggests that establishing an HTD responsible for promoting appropriate clinical use of blood products throughout the hospital may be an effective measure to promote appropriate clinical use of HA. Establishing only an HTD appeared to be insufficient to improve clinical use of HA, but establishing an HTD promoting appropriate clinical use of blood products by introducing GPC of blood products appeared to be a positive measure. Our findings support the international recommendation that a hospital transfusion management system should be established to promote implementation of clinical guidelines on appropriate use of blood products and provide support for policy makers and hospital managers to consider establishing an HTD responsible for promoting appropriate clinical use of HA.

## Data Availability

The datasets generated during the current study are not publicly available due to contracts with the hospitals providing data to the database but are available from the corresponding author on reasonable request.

## Abbreviations

CCI: Charlson Comorbidity Index
DPC: Diagnosis Procedure Combination
GPC: Good practice criteria of blood products
HA: Human albumin
HTC: Hospital transfusion committee
HTD: Hospital transfusion department
ICD-10: International Classification of Diseases and Injuries 10th revision
JCS: Japan Coma Scale
LOS: Length of stay in hospital
MHLW: The Japanese government of Ministry of Health, Labour and Welfare
